# The contribution of discrete-trial naming and visual recognition to rapid automatized naming deficits of dyslexic children with and without a history of language delay

**DOI:** 10.3389/fnhum.2014.00652

**Published:** 2014-09-04

**Authors:** Filippo Gasperini, Daniela Brizzolara, Paola Cristofani, Claudia Casalini, Anna Maria Chilosi

**Affiliations:** Department of Developmental Neuroscience, IRCCS Fondazione Stella MarisPisa, Italy

**Keywords:** dyslexia, RAN, discrete-trial naming, discrete-trial recognition, language delay

## Abstract

Children with Developmental Dyslexia (DD) are impaired in Rapid Automatized Naming (RAN) tasks, where subjects are asked to name arrays of high frequency items as quickly as possible. However the reasons why RAN speed discriminates DD from typical readers are not yet fully understood. Our study was aimed to identify some of the cognitive mechanisms underlying RAN-reading relationship by comparing one group of 32 children with DD with an age-matched control group of typical readers on a naming and a visual recognition task both using a discrete-trial methodology, in addition to a serial RAN task, all using the same stimuli (digits and colors). Results showed a significant slowness of DD children in both serial and discrete-trial naming (DN) tasks regardless of type of stimulus, but no difference between the two groups on the discrete-trial recognition task. Significant differences between DD and control participants in the RAN task disappeared when performance in the DN task was partialled out by covariance analysis for colors, but not for digits. The same pattern held in a subgroup of DD subjects with a history of early language delay (LD). By contrast, in a subsample of DD children without LD the RAN deficit was specific for digits and disappeared after slowness in DN was partialled out. Slowness in DN was more evident for LD than for noLD DD children. Overall, our results confirm previous evidence indicating a name-retrieval deficit as a cognitive impairment underlying RAN slowness in DD children. This deficit seems to be more marked in DD children with previous LD. Moreover, additional cognitive deficits specifically associated with serial RAN tasks have to be taken into account when explaining deficient RAN speed of these latter children. We suggest that partially different cognitive dysfunctions underpin superficially similar RAN impairments in different subgroups of DD subjects.

## Introduction

One of the most robust research findings on the cognitive bases of developmental reading disorders (also known as Developmental Dyslexia, DD) is a deficit of children with DD on rapid serial naming tasks (for reviews see Wolf and Bowers, 1999 and Kirby et al., 2010). The most commonly used measure of this is the rapid automatized naming (RAN) task, in which subjects are presented with arrays of high frequency items (letters, digits, colors, or objects) and asked to name them as quickly as possible. Usually, children with DD perform this task accurately but slowly.

RAN speed deficits in children with DD have been first demonstrated in English speaking readers. However, since early English-based research in the 1970s and 1980s, a strong relation between RAN speed measures and reading acquisition has been documented in a wide array of languages, with both inconsistent (e.g., French: Plaza and Cohen, 2003) and consistent (e.g., German: Wimmer, 1993; Dutch: de Jong and van der Leij, 1999; Finnish: Holopainen et al., 2001; Italian: Di Filippo et al., 2005) alphabetic orthographies.

Despite this evidence, we are currently some way from obtaining a complete understanding of the reasons why RAN performance is related to reading.

Research aiming to reveal the nature of this relationship dates back to the early 80s (for a review see Bowers and Swanson, 1991); from then, many studies have investigated the issue, but evidence is mostly correlational (for reviews see Wolf et al., 2000 and Kirby et al., 2010). Only in the last decade there have been a few studies which experimentally manipulated factors that may account for RAN-reading relationship (Neuhaus and Swank, 2002; Jones et al., 2009; Georgiou et al., 2013; Zoccolotti et al., 2013). Although a consensus begins to emerge for some possible underlying mechanisms, the debate is still largely open for some others.

Substantial agreement exists that post-lexical access articulatory factors do not account for differences on RAN speed among children with different levels of reading ability. For example, when total time to complete RAN tasks is experimentally segregated into a *pause time—*namely the duration of pauses between items in sequenced articulations—and *articulation time*—namely the time to articulate each item—pause time and not articulation time significantly predicts reading ability, in both normal and DD readers (Georgiou et al., 2006; Araújo et al., 2011b). Hence, these results point to the inter-item processing—namely the processes intercurring from attentional disengaging from one stimulus to name retrieval for the next one—as the *locus* that drives RAN-reading association.

According to one influential view, RAN should be considered as part of a larger phonological construct together with phonological awareness (PA) and phonological short-term memory (PSTM), in that it primary reflects the rate of access to and retrieval of stored phonological information in long-term memory (Wagner et al., 1993; Pennington et al., 2001; Chiappe et al., 2002). Evidence supporting a role of phonological lexical access in mediating the RAN-reading relationship comes from studies using a discrete-trial methodology. These studies have often found that latency to name singularly presented highly familiar stimuli (the same used in the serial RAN tasks) is significantly correlated with reading measures, in both samples of children unselected for reading skill (Levy and Hinchley, 1990) and children with DD (Bowers and Swanson, 1991; Fawcett and Nicolson, 1994; Chiappe et al., 2002; Jones et al., 2009).

However, not all authors consider RAN performance as just another instance of phonological processing ability together with PA and PSTM. For example, Wolf and Bowers (1999) emphasize the multi-componential nature of serial RAN tasks, placing stronger emphasis on the efficiency with which multiple processes (attentional, visual, phonological, semantic and articulatory) are integrated through precise timing mechanisms, which in turn calls into question general processing speed. As a consequence, Wolf and Bowers (1999) state that phonological processes—which they index essentially through PA—and mechanisms underlying RAN performance represent two independent sources of variability in reading ability.

A good deal of evidence supports Wolf and Bowers’ position. For example, RAN and PA are not strongly correlated (Swanson et al., 2003). Moreover, many cross-sectional and longitudinal studies have found that RAN speed contributes uniquely to the variance in reading ability when PA is controlled (Torgesen et al., 1997; Parrila et al., 2004; Kirby et al., 2009). However, PA does not end all of phonological processing; in their empirical model of phonological processing, Wagner et al. (1993) identified three significantly correlated but distinct factors: PA (blending and segmenting sounds from words), PSTM (digit span), and lexical access (naming speed). Therefore, the fact that RAN speed and PA are only modestly correlated and each predicts a unique proportion of variance in reading is not in conflict with the hypothesis that phonological processing abilities, in particular access to and retrieval of phonological codes from long-term memory, contribute substantially to RAN-reading relationship.

Other possible cognitive mechanisms underlying RAN speed and mediating its relationship with reading include early visual processes. RAN efficiency might reflect both the initial ability to visually analyze the stimuli’s constituent features and the subsequent proficiency to integrate visual pattern information with stored representations. The role of visual processing in RAN-reading relationship has so far received minor attention and remains ill understood. Some indirect evidence seems consistent with the hypothesis of a contribution of low-level visual factors to RAN performance and its association with reading (Stainthorp et al., 2010; Araújo et al., 2011a). Using a visual naming task Araújo et al. (2011a) manipulated some variables related to early stages of visual processing of objects and found that, in contrast to control readers, naming performance of DD subjects did not improve with color or 3-dimensional object presentation compared with black-and-white or 2-dimensional object presentation respectively. These results lead the authors to state that “*processes involved in early visual feature analysis or in the integration of visual information stored in long-term memory might be affected in dyslexia*” (p. 224). However, directly comparing the contribution of a RAN task and a visual search task using the same stimulus materials, Di Filippo et al. (2006) found that the disadvantage of DD children with respect to controls on RAN tasks remained unchanged when the visual search performance was partialled out by covariance analysis. These latter findings are at odds with a significant role of early visual processing in driving RAN-reading relationship.

Further explanations of RAN-reading relationship arise from experimental evidence that serial RAN tasks are usually more closely correlated with reading than discrete-trial RAN paradigms (Bowers, 1995; Pennington et al., 2001; Jones et al., 2009; Logan et al., 2011). One interpretation emphasizes the importance of sequential requirements of RAN tasks as a way of explaining their relation with reading, as it is reasonable that speeded naming of multiple items in a matrix format requires both inhibition of previous (already named) stimuli and efficient processing of upcoming items (Jones et al., 2009). Actually, recent findings indicate that both foveal and peripheral processing occur while performing RAN tasks as well as in reading. Recent experimental evidence indicates that children with DD have difficulty not only distinguishing between multiple competing phonological representations at foveal stages of processing (when a verbal response is required), but also between multiple activated orthographic codes during parafoveal processing (Jones et al., 2013). More generally, RAN and reading could be related because several items are visible at once in both tasks, allowing subjects to pre-process some items in parafovea. Studies on text reading have shown that for typical readers the availability of upcoming words in the parafovea increases the speed with which the text is read (Sereno and Rayner, 2000); however, parafoveal information may in fact act as a source of interference for children with DD, in both reading (Chace et al., 2005; Pernet et al., 2006) and RAN tasks (Jones et al., 2009).

Overall, despite a growing number of studies in the last three decades, cognitive mechanisms underlying RAN-reading relationship are still a matter of considerable debate. It should also be considered that processes mediating RAN-reading relationship may at least partially change with reading development, when a progressive shift occurs from a serial grapheme-phoneme decoding strategy toward a parallel “sight-word” reading. Recent data on Greek speaking children (Protopapas et al., 2013) support this hypothesis by showing that the amount of common variance between serial RAN and reading was mostly explained by discrete-trial naming (DN) in 2nd graders, while a stronger contribution from sequential processing was evident among 6th graders. Based on these results, it is plausible to expect that processes driving RAN-reading relationship may also be different between normally developing and DD readers.

The general aim of the present study was to give a contribution in identifying cognitive mechanisms underlying RAN-reading relationship in children learning Italian, a language characterized by a transparent orthography. In a previous study, we obtained evidence that RAN and not phonological processing abilities (as assessed through PA and PSTM tasks) may represent the main cognitive marker of DD in Italian children (Brizzolara et al., 2006). This evidence is consistent with a growing body of studies in which RAN speed has been shown to be a strong predictor of reading ability in orthographically transparent languages, both in sample of children unselected for reading ability and in children with reading disability (e.g., for Finnish: Holopainen et al., 2001; for German: Landerl, 2001; for Dutch: de Jong and van der Leij, 2003; for Greek: Papadopoulos et al., 2009). Because of the relevance of cognitive processes underlying RAN speed for reading ability in transparent orthographies, a deeper understanding of mechanisms driving RAN-reading relationship seems of particular relevance.

In the present study, we focused on some of the cognitive processes underpinning RAN speed which may differentiate between children with DD and average readers, in particular, visual recognition of single items, lexical access and sequential processing of multiple items.

Firstly, we compared a group of DD children with an age-matched control group of typical readers on a DN paradigm in which items were digits or colors.

Secondly, children with DD and their controls were contrasted on a motor choice-reaction time task using the same stimuli as in the DN, in which subjects had to discriminate between a target stimulus and four distracters. Such experimental manipulation at the output stage of DN removes much if not all the name-retrieval component, allowing a direct test for the role of early visual processes involved in naming of isolated items. To our knowledge this is the first attempt to investigate the potential contribution of early visual factors to RAN-reading relationship using the same material as the serial RAN task, with specific focus on single-item processing level. Di Filippo et al. (2005, 2006) had previously made a similar experimental manipulation, using a visual search task with the same stimulus material as RAN; however, in Di Filippo et al.’s studies control on early visual processing was more wide-ranging (including scanning of the stimuli) and less specifically focused on single stimulus identification.

At the same time, investigating both DN and recognition in the same subjects using the same stimulus material allows testing the role of lexical access in differentiating RAN performance of DD and average readers, once potential differences at the stage of visual identification have been removed.

A further step of our study was to compare children with DD and controls on a serial RAN paradigm using the same stimuli as in the other two tasks, to verify if the expected differences in serial RAN survived after statistical control for the possible significant effects of both DN and discrete recognition (DR). If serial RAN continues to discriminate between children with DD and controls, then a further contribution to RAN-reading relationship has to be probably found in cognitive factors outside single-item processing (such as sequencing of multiple items and/or higher demands on precise timing mechanisms posed by serial RAN).

In our study children with DD were further assigned to two sub-groups according to whether or not they had a history of early language delay (LD) as determined retrospectively by parental report. In previous work we demonstrated a somewhat different cognitive profile in Italian DD children with and without a history of LD (Brizzolara et al., 2006; Chilosi et al., 2009; Pecini et al., 2011). In fact, while both groups shared a comparable RAN speed deficit, only DD children with a previous LD showed a moderate but widespread verbal deficit, extending from phonological processing (PA and PSTM) to other components of linguistic processing (lexical phonology, semantics and syntax). However, as the classification of LD in children with DD is based on a retrospective criterion (i.e., based on parents’ report), in the present study a set of standardized behavioral and cognitive tests was also administered. These included measures of sub-lexical and lexical reading, written text comprehension, PSTM, verbal semantic knowledge and vocabulary. We expected to find lower scores among DD children with LD than among DD children without LD (LD-DD and noLD-DD, respectively) in all the oral verbal measures and in the test of reading comprehension, consistently with previous evidence indicating a moderate but widespread verbal deficit (i.e., not limited to phonological processing) among LD-DD children that does not affect noLD-DD subjects (Chilosi et al., 2009).

There is now mounting evidence that DD is an heterogeneous neurobiological condition (Eckert, 2004; Jednoróg et al., 2013) associated with multiple impairments in different cognitive domains (Bosse et al., 2007; Menghini et al., 2010), including phonological processing (Vellutino et al., 2004), early visual analyses (Stein, 2001; Martelli et al., 2009), skills automatization (Nicolson et al., 2001) and visual-spatial attention (Hari and Renvall, 2001; Franceschini et al., 2012). As a consequence, it would not be surprising if different cognitive deficits underpin impaired RAN performance in subgroups of DD children with distinct neurocognitive phenotypes.

On the basis of the reviewed evidence, we expected that children with DD considered as a group would be significantly slower than chronological age-matched controls on both serial RAN and DN tasks. We also expected that differences between DD and control groups on serial RAN tasks survived statistical control for differences in DN tasks. Both expectations seemed plausible in children learning a transparent orthography, as Italian, in which DD has been mainly characterized by a reading fluency deficit in the face of rather accurate decoding (for a review see Wimmer and Schurz, 2010; see also Zoccolotti et al., 1999 for evidence in Italian). The characteristic reading speed deficit of these children might be, in fact, equally well explained by a deficient orthography to phonology mapping at both sub-lexical and lexical level of reading, indexed by impaired DN (de Jong, 2011), as well as by a reduced ability to simultaneously process multiple adjacent items in the written text, tapped by the unique contribution of serial RAN to reading difficulties (Protopapas et al., 2013).

Predictions about DR tasks in our study were more open due to the variable results reported in the literature.

Hypotheses on possible different cognitive mechanisms underlying RAN-reading relationship in LD-DD and noLD-DD were more speculative, as to our knowledge this is the first study to address such topic. One possibility is that impaired lexical access is particularly relevant for RAN speed deficits in LD-DD children. Although name-retrieval deficits have often been described in both DD subjects without apparent previous or concurrent language difficulties (Wolf and Obregon, 1992; Swan and Goswami, 1997; Hanly and Vanderberg, 2010) and in children with specific oral language difficulties (also known as Specific Language Impairment, SLI; Kail and Leonard, 1986; Befi-Lopes et al., 2010; Coady, 2013), word finding difficulties might be more pronounced in LD-DD children in comparison with noLD-DD children: the two groups might share a common phonological lexical access deficit, while only the former would show an additional semantic deficit. Indeed, evidence exists for both a phonological (Coady, 2013) and a semantic (Kail and Leonard, 1986; Befi-Lopes et al., 2010) account of naming difficulties in children with oral language difficulties.

If impaired lexical access is particularly relevant for RAN speed deficits in LD-DD children, then slower performance on the DN tasks should be more evident in these subjects than in noLD-DD children. However, this might be true for the color more than for the digit condition. In fact, conceptual activation would mediate mainly color naming (Heurley et al., 2013), while more direct arbitrary mappings from visual stimuli to phonological labels occur for digits (Manis et al., 1999). Possibly, the unique contribution of serial RAN tasks over the one played by DN tasks would be smaller in LD-DD than in noLD-DD subjects.

## Materials and methods

### Participants

Informed consent was obtained from all the parents of participants in compliance with the Helsinki Declaration.

Participants with DD were selected on the basis of consecutive referrals to the Division of Child Neurology and Psychiatry of the University of Pisa from May 2009 to October 2010 for suspected specific learning disabilities. Criteria for inclusion in the DD sample were the following:

- non-verbal intelligence within normal limits, as assessed by Raven’s Colored Progressive Matrices (Raven, 1984) for children from third to fifth grade and by Picture Completion and Block Design subtests of WISC-III (Wechsler, 1991) jointly considered for children from sixth to eighth grade (see below, for details);
- impaired score on a standardized single words reading test from the DDE-2 (Battery for the Evaluation of DD and Dysorthography), 2nd edition (Sartori et al., 2007) (see below);
- regular school attendance;
- absence of neurological abnormalities on a standard neurological examination;
- normal or corrected to normal visual acuity;
- no clinical evidence of oral language impairment at the time of assessment. Assessment of oral language was carried out by a child neuropsychiatrist with special expertise in speech and language disorders (A.C.) using a semi-structured interview. Normal fluency, well-formed sentences and the absence of phonological, semantic and grammatical errors in conversation were considered as signs of adequate language organization.

Thirty two (18 males and 14 females) children fulfilled these criteria. The mean age for children with DD was 11 years and 2 months, with a standard deviation (SD) of 1 year and 9 months. The youngest children were second graders and the oldest were eighth graders. More specifically, 15 children attended Primary school (from second grade to fifth grade) while 17 children attended Secondary school (from sixth to eighth grade). All DD participants were Italian native speakers. No child had been diagnosed as Attention Deficit Hyperactivity Disorder (ADHD) at the time of assessment.

Each child’s clinical history was investigated by means of an assessment interview with his or her parents; this was carried out by the same child neuropsychiatrist with special expertise in speech and language disorders (A.C.) who assessed oral language abilities of children. The parents were also asked to fill out a questionnaire (Brizzolara et al., 2006; Chilosi et al., 2009) on motor, cognitive, and language developmental milestones. In order to encourage the parents to recall basic language milestones, examples of typical children’s utterances were provided. A child was considered to have a positive history of LD if the analysis of his/her questionnaire revealed at least one of these signs: (1) no vocabulary burst before 24 months; (2) late combinatory use of words, that is, after 30 months; (3) persistence of grammatically incomplete sentences after 4 years of age; and (4) persistence of phonological mispronunciations after 4 years of age. On the basis of these criteria 18 children (11 males, 7 females) were considered as having had a LD. They had a mean age of 11 years and 1 month (SD = 2 years and 4 months). No language delay (noLD) was documented retrospectively in 14 children (7 males and 7 females). Their mean age was 11 years and 5 months. No significant difference for age (*F*_(1,30)_ = 0.21, *ns*) was present between LD and noLD DD groups.

One group of 32 Italian, native-speaking typical readers served as control for the children with DD. These participants were selected from several Primary and Secondary public schools and individually matched with DD subjects by sex and chronological age (± 3 months). For the children to be included, they also had to perform normally on the same standardized single word reading test (see below) and non-verbal intelligence tests used in children with DD. The mean age of the control group was 11 years and 2 months (SD = 1 year and 8 months), a value not significantly different from that of the DD group as expected on the basis of the selection criterion (*F*_(1,62)_ = 0.01, *ns*).

Control children were further subdivided into two groups, in which subjects were matched individually with DD children of LD and noLD group respectively. As expected, each DD group did not differ significantly from its own control group for age (*F*_(1,34)_ = 0.06 for the comparison involving the LD children and *F*_(1,26)_ = 0.00 for the comparison with the noLD children, both *ns*).

### Materials and procedure

#### Non-verbal intelligence

Non-verbal intelligence was assessed by Raven’s Colored Progressive Matrices (Raven, 1984; Italian standardization, Pruneti et al., 1996) for children from third to fifth grade and by Picture Completion and Block Design subtests of WISC-III (Wechsler, 1991; Italian standardization Orsini and Picone, 2006) jointly considered for children from sixth to eighth grade, in both the DD and control samples.

The cut-off for non-verbal intelligence within normal limits was set at one SD below the mean of the normative sample for the appropriate age level; for children from sixth to eighth grade the average between the Picture Completion and Block Design scaled scores was computed.

Mean *z* score (and SD) for non-verbal intelligence of the DD and control groups are reported in Table 1. The two groups did not differ significantly for non-verbal intelligence (*F*_(1,62)_ = 0.16, *ns*).

**Table 1:** **Means and SDs (in brackets) of the whole DD sample, of the sub-groups of DD participants with (LD) and without (noLD) a history of language delay and the respective control groups on non-verbal intellective level and single word reading measures**.

	DD subjects (whole sample, *n* = 32)	Controls (whole sample, *n* = 32)	LD-DD subjects (*n* = 18)	Controls for the LD-DD subjects (*n* = 18)	noLD-DD subjects (*n* = 14)	Controls for the noLD-DD subjects (*n* = 14)
Non-verbal intellective level (*z* scores)	00.12 (0.75)	0.19 (0.54)	00.02 (0.76)	0.22 (0.59)	00.26 (0.73)	0.15 (0.50)
Single word reading Accuracy (*z* scores)	−4.85 (8.99)	0.29 (0.73)	−6.33 (11.50)	0.45 (0.70)	−2.79 (2.68)	0.07 (0.74)
Single word reading Speed (*z* scores)	−6.27 (7.80)	0.36 (0.70)	−7.49 (10.05)	0.38 (0.78)	−4.57 (1.94)	0.33 (0.60)

*z scores for both non-verbal intelligence level and reading measures were computed according to the corresponding normative data for age (non-verbal intelligence level) or grade level (reading measures; for details see the text)*.

Likewise, no differences existed in non-verbal intelligence between the DD groups and their control subgroups (*F*_(1,34)_ = 0.77 for the comparison involving LD children and* F*_(1,26)_ = 0.20 for the comparison with the noLD children, both *ns*), as well as between the LD-DD and the noLD-DD subjects (*F*_(1,30)_ = 0.83, *ns*).

Mean *z* scores (and SDs) for non-verbal intelligence of the DD and control subgroups are reported in Table 1.

#### Reading assessment

Reading level for inclusion in the DD or in the control group was assessed using one subtest from the Battery for the Evaluation of DD and Dysorthography, 2nd Edition (Sartori et al., 2007). In this subtest subjects have to read aloud as quickly and accurately as possible four lists of 28 words with high or low frequency (4- to 8-letter long). Total number of errors and speed of reading (syllables/second) are computed. Raw scores were converted to *z* scores according to standard reference data; normative data are available separately for each grade from second to eighth grade.

A *z* score lower than 2 with respect to the mean of the normative sample for either accuracy or speed was taken as the cut-off criterion for inclusion in the DD group. This disjunctive criterion was used because it has been shown that subjects with DD can flexibly adapt their speed-accuracy rate (Hendriks and Kolk, 1997); consequently, a selection based on both parameters might fail to detect selective cases of pathological performance. At the same time, to limit overlaps between the DD and the control group, we adopted a conservative criterion: for children to be included in the control group the performance in the reading test could not be lower than one SD below the mean of the normative sample for either accuracy or speed.

Mean (and SD) *z* scores for the reading measures of both the DD and the control group are reported in Table 1. The control readers’ performance was close to zero for both accuracy and speed. On the contrary, DD readers performed very poorly on both parameters. As expected on the basis of selection criteria, DD children scored significantly worse than control readers on both accuracy (*F*_(1,62)_ = 10.05, *p* < 0.01) and speed (*F*_(1,62)_ = 22.18, *p* < 0.001). Given the heterogeneity of age in our sample, we compared Primary with Secondary school children in both speed and accuracy raw scores of single-word reading. No significant difference emerged for both measures (*F*_(1,30)_ = 1.49, *p* = 0.23, *ŋ*^2^ = 0.05 for speed; *F*_(1,30)_ = 1.64, *p* = 0.21, *ŋ*^2^ = 0.05 for accuracy).

Table 1 also reports the mean (and SD) *z* scores for the reading measures of both LD- and noLD-DD children and the corresponding control children. No difference emerged between LD and noLD groups on accuracy (*F*_(1,30)_ = 1.59, *ns*) and speed (*F*_(1,30)_ = 1.46, *ns*). On the contrary, as expected, both DD samples differed significantly from the corresponding control group on both the reading parameters (as regards LD children: *F*_(1,34)_ = 6.25, *p* < 0.05 and *F*_(1,34)_ = 11.02, *p* < 0.01 for accuracy and speed respectively; as regards noLD participants: *F*_(1,26)_ = 13.90, *p* < 0.01 and *F*_(1,26)_ = 76.09, *p* < 0.001 for accuracy and speed, respectively).

#### Other literacy measures

Reading decoding abilities were further investigated by means of a test of non-word reading, which is usually considered to tap the sub-lexical reading route. Non-word reading was assessed with a subtest from the same standardized battery of the word reading test (Sartori et al., 2007). In this subtest subjects have to read aloud as quickly and accurately as possible three lists of 16 non-words (5 to 9-letter long) in line with the phonotactic and phonographic rules of the Italian language. Total number of errors and speed of reading (syllables/second) are computed. Raw scores were converted to *z* scores according to standard reference data; normative data are available separately for each grade from second to eighth grade.

Text reading comprehension was also investigated, using a standardized test from the MT Reading battery (Cornoldi and Colpo, 1995, 1998). A meaningful passage is presented without a time limit. The participant has to read it silently and respond to multiple-choice questions. Stimulus materials, number of questions (10 to 15) and related reference norms vary from school level. Raw scores were converted to *z* scores according to standard reference data.

Mean (and SD) *z* scores for the non-word reading and written text comprehension measures of both the DD groups are reported in Table 2. The two groups did not differ significantly on non-word reading (*F*_(1,30)_ = 2.03, *ns* and *F*_(1,30)_ = 1.07, *ns* for accuracy and speed, respectively). A significant difference emerged for text reading comprehension (*F*_(1,30)_ = 4.95, *p* < 0.05), with a lower performance from LD-DD than noLD-DD children. In absolute terms, the mean performance of the former subgroup was more than one SD below the mean of the normative sample.

**Table 2:** **Means and SDs (in brackets) of sub-groups of DD participants with (LD) and without (noLD) a history of language delay on literacy (non-word reading and written text comprehension) and oral verbal measures**.

			LD-DD subjects (*n* = 18)	noLD-DD subjects (*n* = 14)
Literacy measures	Non-word reading (*z* scores)	Accuracy	−3.40 (2.96)	−2.14 (1.30)
		Speed	−2.96 (3.67)	−4.15 (2.11)
Oral verbal measures	Written text comprehension (*z* scores)		−1.17 (1.25)	−0.23 (0.90)
	Phonological STM (*z* scores)		−1.30 (0.84)	−0.50 (0.77)
	WISC-III Information (scaled scores)		006.30 (2.98)	09.00 (1.58)
	WISC-III Vocabulary (scaled scores)		008.42 (2.43)	09.70 (2.31)

*z scores for the literacy and Phonological STM measures and scaled scores for the WISC-III subtests were computed according to corresponding normative data for grade level (reading measures) or age (Phonological STM and WISC-III subtests) (for details see the text)*.

#### Oral verbal measures

##### Phonological short-term memory (PSTM)

PSTM abilities were examined with a shortened version of a word span test (Ferretti et al., 2003), requiring the child to repeat two lists of Italian high-frequency, disyllabic words varying for phonological similarity. Stimulus presentation is controlled by a PC using a dedicated software. For each list (with or without phonological similar words), sequences of increasing length are presented (from two to seven words). The child is required to repeat the words in the correct order. The list presentation is interrupted when the child fails on three out of five series of the same length. For each list, memory span is the number of words of the longest sequence correctly repeated at least in three out of five presentations. The raw scores were converted to *z* scores according to standard reference data of the test, separately for each list, and then averaged to get a single *z* score for each subject.

Table 2 shows the means and SDs for the PSTM task of both LD-DD and noLD-DD group.

PSTM was significantly lower in the LD than in the noLD-DD children (*F*_(1,30)_ = 7.44, *p* < 0.05), falling more than one SD below the mean of the normative sample in the former.

##### Verbal semantics

Verbal semantic knowledge was investigated with the WISC-III Information sub-test (Wechsler, 1991; Orsini and Picone, 2006). Raw scores were transformed into scaled scores on a 1–19 scale, with mean = 10 and SD = 3. Data for this test were available for 28 children with DD of our sample (15 LD and 13 noLD; see Table 2). The two groups differed significantly on this subtest (*F*_(1,26)_ = 5.86, *p* < 0.05): LD-DD children performed worse than noLD-DD children, scoring as a group more than one SD below the population mean.

##### Vocabulary

Word knowledge (and also verbal concept formation) was examined using the WISC-III Vocabulary sub-test (Wechsler, 1991; Orsini and Picone, 2006). Raw scores were transformed into scaled scores on a 1–19 scale, with mean = 10 and SD = 3. Data for this test were available for 28 children with DD of our sample (15 LD and 13 noLD; see Table 2). The LD children performed somewhat lower than the noLD-DD subjects on the Vocabulary sub-test, but the difference was not significant (*F*_(1,26)_ = 1.59, *ns*).

#### Experimental tasks

##### RAN test

###### Stimuli

For the RAN (or serial rapid naming) tasks, materials were adapted from Di Filippo et al. (2005, 2006). Stimuli were matrices of digits or colors on a white background. In each condition, five different stimuli were presented. The digits were 2, 4, 6, 7, and 9, generated with Helvetica font (size 24) and black typed. The colors were presented in small 1 by 1 cm squares; they were black, blue, red, yellow, and green. There were five rows of 10 stimuli in each matrix for a total of 50 stimuli. There was one matrix for each condition. A smaller matrix with two rows of five stimuli was also created for both digit and color condition to be used in a practice trial (see below). Target sequence was randomized within each matrix.

###### Procedure

Stimuli were displayed on a PC screen. The child was requested to name each stimulus in the matrix as quickly and accurately as possible, working from left to right and from top to bottom. A practice session with a small (10 stimuli) matrix was run for each condition; during this session, the examiner corrected any errors made by the child. For each condition (numbers or colors) time to complete the task was measured using a stopwatch and total time in seconds was used as the dependent measure. Naming errors were also recorded.

##### Discrete-trial naming (DN) test

###### Stimuli

Stimuli were the same as in the RAN test, except that in the DN tests both number and colors appeared singly on the PC screen on a white background. Target sequence was randomized for each condition. Similarly to the RAN task, for each condition (numbers or colors) a total of 50 stimuli were presented within a single block.

###### Procedure

For each condition (numbers and colors), stimuli were displayed singly in the center of a PC screen. Presentation was controlled by SuperLab Pro 2.0 package (Cedrus Corporation, 2002; San Pedro, California). The child was asked to name each digit or color as fast and accurately as possible. The stimulus remained on the screen until the subject responded or for a time limit of 6000 ms. Then a 500 ms blank followed before the next stimulus appeared. In order to avoid false responses, subjects were explicitly requested not to self-correct if they realized a naming error had occurred. For each condition, participants were given a practice session with 10 stimuli; at the end of this session children were corrected for any naming errors and taught to avoid self-corrections. Vocal latencies were recorded using the SuperLab Pro 2.0 package. Naming errors were also computed. In a few instances, trials were not valid due to technical failures or false responses; more generally, all latencies under 250 ms were counted as invalid trials. Only latencies for valid and correct trials were analyzed. For each condition the median response latency and the total number of naming errors were computed. Median latency was chosen as measure of central tendency to remove the influence of outlier values.

##### Discrete-trial recognition (DR) test

###### Stimuli

Stimuli and format presentation were the same as in the DN test. Target sequence was randomized for each condition, but the order of presentation was different than in the DN test.

###### Procedure

For each stimulus the trial sequence and temporal parameters were the same as in the DN test. The stimulus was singly displayed at the center of the PC screen and disappeared with the subject’s response or after a time limit of 6000 ms. Then, a 500 ms blank followed before the next stimulus was presented. A motor choice-reaction time task was used to estimate efficiency of visual processing underpinning single-item naming. Participants were asked to press one of two keys on a response pad as fast and accurately as possible when the target stimulus appeared (number 7 for the digit condition and a green square for the color condition) and to press the other key when all the other stimuli were displayed. Responses to the target were made using the index finger of the dominant hand, while those to the other stimuli with the index finger of the non-dominant hand. Instructions were given to keep both index fingers on the corresponding keys for the entire session. For each condition, a practice session with 10 stimuli was given, in which participants’ errors were corrected.

Both latency and accuracy of response were recorded by SuperLab Pro 2.0 for each stimulus. Latencies under 150 ms were considered as invalid trials, as they could be either technical failures or anticipations. Only latencies for valid or correct trials were analyzed. For each condition both median response latency and total number of recognition errors were computed.

#### General procedure

Participants were tested individually in a quiet room, located at the Division of Child Neurology and Psychiatry of Pisa University for children with DD or at their own school for control children. All the tests were administered in a single session. Each session started with the non-verbal intelligence tests, followed by the reading test and then by the experimental tasks. For each group, presentation sequence of the three experimental tasks was counterbalanced across participants according to a 3 × 3 Latin Square design. Likewise, for each experimental task the order of the two conditions (digits and colors) was counterbalanced across participants using a 2 × 2 Latin Square design.

### Data analysis

A first series of analyses compared the performances of control and DD subjects considered as one group on the RAN, DN and DR tests, separately for each condition (digits or colors).

Accuracy was very high for every task and condition, for both control and DD children. The mean percentages of errors are presented in Table 3 for all three tasks according to condition (digits and colors) separately for control and DD subjects. Even in the DR tests where level of accuracy was slightly lower, percentage of errors was always below 5% and not statistically different between DD and control children (*F*_(1,62)_ = 1.85 for comparison on DR of digits and *F*_(1,62)_ = 0.15 for comparison on DR of colors, both *ns*). As a consequence, for each experimental task statistical comparisons were restricted to time measures. Mean total naming times in RAN tests and median response latencies in DN and DR tests were submitted to a one-way ANOVA with group (DD children, controls) as unrepeated factor. ANOVAs were carried out separately for digits and colors in each experimental task. In order to assess equality of variances between DD and control children the Levene’s test was run for each comparison we made. In no case, variances differed significantly between groups (all *p* > 0.05).

**Table 3:** **Mean percentages of errors and SDs (in brackets) of the whole DD sample, of the sub-groups of DD participants with (LD) and without (noLD) a history of language delay and the respective control groups on the three experimental tasks as a function of type of stimulus (digits and colors)**.

	DD subjects (whole sample, *n* = 32)	Controls (whole sample, *n* = 32)	LD-DD subjects (*n* = 18)	Controls for the LD-DD subjects (*n* = 18)	noLD-DD subjects (*n* = 14)	Controls for the noLD-DD subjects (*n* = 14)
RAN of digits	1.69 (3.67)	0.19 (0.59)	1.00 (2.84)	0.34 (0.76)	2.58 (4.46)	0.00 (0.00)
RAN of colors	2.81 (4.06)	1.37 (2.06)	3.10 (5.00)	0.66 (1.68)	2.42 (2.50)	2.28 (2.20)
DN of digits	0.50 (1.61)	0.12 (0.71)	0.78 (2.08)	0.00 (0.00)	0.14 (0.54)	0.28 (1.06)
DN of colors	1.75 (2.58)	0.50 (1.13)	2.34 (3.16)	0.34 (0.76)	1.00 (1.30)	0.72 (1.48)
DR of digits	4.00 (2.69)	3.06 (2.83)	3.78 (2.36)	3.22 (2.84)	4.28 (3.12)	2.86 (2.90)
DR of colors	4.69 (3.58)	4.31 (4.04)	3.88 (2.70)	5.22 (4.30)	5.72 (4.36)	3.14 (3.48)

For each comparison between DD and control groups effect size was also calculated by computing the Eta squared (*ŋ*^2^) value, which indicates the proportion of variance in the dependent variable explained by the independent variable (the reading group in our case).

As significant differences between the two groups on both RAN and DN tests emerged, an ANCOVA on mean RAN times using DN median latencies as covariates was carried out in order to determine the possible modulating role of the performance on the DN tests over the RAN tests. Two separate ANCOVAs were run for digit and color conditions.

In a second series of analyses the same statistical techniques were used to compare, separately, both LD- and noLD-DD children with their own control subgroups. As in the whole DD sample, percentages of errors (see Table 3) were very low for all three experimental tasks in both DD subgroups; it was slightly higher for the DR tasks, although always under 6% for both groups and with no significant differences from controls for both LD-DD group (*F*_(1,34)_ = 0.41 and *F*_(1,30)_ = 1.24 for digits and colors respectively, both *ns*) and noLD-DD group (*F*_(1,26)_ = 1.57 and *F*_(1,26)_ = 2.97 for digits and colors respectively, both *ns*). As a consequence, accuracy was not further examined. Time measures of each experimental task were submitted to two separate one-way.

ANOVAs with group (either LD- or noLD-DD *vs*. respective controls) as unrepeated factor. When the Levene’s test for equality of variances between groups was applied, for only 1 out of 12 comparisons (the one on RAN of digits for LD-DD *vs*. control children) variances significantly differed between groups (*F* = 5.19, *p* < 0.05); in this case, violation of equality of variances was corrected using the Welch-Satterthwaite method. Effects sizes using *ŋ*^2^ value were also computed for the different comparisons between LD and noLD DD children and respective controls.

Again, as both RAN and DN time measures were significantly different in most cases, when necessary mean RAN times were submitted to ANCOVAs with group (either LD- or noLD-DD children *vs*. respective controls) as unrepeated factor and DN median latencies as covariates, separately for the digit and color condition, to determine if differences on RAN performance survived when DN performances were partialled out.

Finally, one-way ANOVAs were carried out to directly compare LD- and noLD-DD children on the three experimental tasks. Effects sizes using *ŋ*^2^ were calculated for each comparison.

For all statistical analyses significance level was set at *p* < 0.05.

## Results

### Whole DD sample

Means (and SDs) for the three experimental tasks of both the whole DD sample and control group are reported in Table 4 according to type of stimulus.

**Table 4:** **Means and SDs (in brackets) of whole DD sample, of the sub-groups of DD participants with (LD) and without (noLD) a history of language delay and the respective control groups on the three experimental tasks as a function of type of stimulus (digits and colors)**.

	DD subjects (whole sample, *n* = 32)	Controls (whole sample, *n* = 32)	LD-DD subjects (*n* = 18)	Controls for the LD-DD subjects (*n* = 18)	noLD-DD subjects (*n* = 14)	Controls for the noLD-DD subjects (*n* = 14)
RAN of digits (s)	32.72 (8.79)	22.50 (6.50)	33.39 (7.92)	21.39 (4.53)	31.86 (10.04)	23.93 (8.38)
RAN of colors (s)	43.69 (12.35)	35.44 (9.52)	46.00 (12.94)	34.44 (9.10)	40.71 (11.30)	36.71 (10.23)
DN of digits (ms)	686.5 (120.3)	544.2 (88.2)	713.7 (103.9)	529.2 (83.1)	651.5 (134.3)	563.4 (93.8)
DN of colors (ms)	804.6 (141.2)	650.0 (113.9)	818.5 (143.9)	627.4 (123.2)	786.6 (140.9)	679.1 (97.3)
DR of digits (ms)	452.5 (136.1)	420.5 (124.9)	448.7 (118.0)	403.4 (98.9)	457.4 (161.1)	442.5 (153.2)
DR of colors (ms)	449.9 (111.8)	432.9 (99.3)	443.9 (106.4)	407.2 (72.9)	457.7 (121.9)	466.0 (120.2)

ANOVA on RAN times revealed a significant group effect, for both digits (*F*_(1,62)_ = 27.95, *p* < 0.001) and colors (*F*_(1,62)_ = 8.96, *p* < 0.01): DD children were significantly slower than controls in both RAN of digits and colors. Effect size was very large for digits (*ŋ*^2^ = 0.31, that is 31% of RAN times for digits explained by reading group) and medium-high for colors (*ŋ*^2^ = 0.13).

A significant group effect emerged also for DN response latencies, regardless of type of stimulus: latencies to respond were higher in DD group than in control group, for both digits (*F*_(1,62)_ = 29.16, *p* < 0.001) and colors (*F*_(1,62)_ = 23.22, *p* < 0.001). For both types of stimulus effect size was very large (*ŋ*^2^ = 0.32 and *ŋ*^2^ = 0.27 for digits and colors respectively).

On the contrary, no statistical difference resulted in DR response latencies between DD subjects and normal readers. This was true for both digits (*F*_(1,62)_ = 0.96, *ns*) and colors (*F*_(1,62)_ = 0.41, *ns*). Effect size was very small for both types of stimuli (*ŋ*^2^ = 0.01 in both conditions). It should be noted that DD and control participants did not differ also for number of errors on DR tasks (see above Section on Data Analyses); then a speed-accuracy trade-off on these tasks in the DD sample is to be excluded.

Results of ANCOVA on RAN times using DN response latencies as covariates gave different results for the two types of stimulus. When DN response latencies were partialled out, the group effect on RAN times remained statistically significant for digits (*F*_(1,62)_ = 6.81, *p* = 0.01) but not for colors (*F*_(1,62)_ = 0.26, *ns*), with effect size medium for the former (*ŋ*^2^ = 0.10) and negligible for the latter (*ŋ*^2^ = 0.00).

### LD-DD sample

Table 4 shows means and SDs for all experimental tasks of both LD-DD children and respective controls according to type of stimulus.

LD-DD participants and their controls differed significantly on RAN speed for both digits (*F*_(1,34)_ = 31.14, *p* < 0.001) and colors (*F*_(1,34)_ = 9.60, *p* < 0.01) with large effect sizes of the group factor for both types of stimulus (*ŋ*^2^ = 0.48 and *ŋ*^2^ = 0.22 for digits and colors, respectively).

Significant differences between the groups were also evident on the DN response latencies, regardless of type of stimulus: mean response latencies in DN tasks were higher in LD-DD children than in typically developing readers for both digits (*F*_(1,34)_ = 34.60, *p* < 0.001) and colors (*F*_(1,34)_ = 18.32, *p* < 0.001). In both conditions effect sizes were very large (*ŋ*^2^ = 0.50 and *ŋ*^2^ = 0.35 for digits and colors, respectively).

By contrast, no significant group effect was evident on DR response latencies. This applied to both digit (*F*_(1,34)_ = 1.56, *ns*) and color condition (*F*_(1,34)_ = 1.44, *ns*). Effect size was small for both types of stimuli (*ŋ*^2^ = 0.04 for both digits and colors). As already reported, also accuracy level did not differ between LD-DD children and their controls on DR tasks.

When the ANCOVA was performed on RAN times with DN latencies as covariates, a significant group effect was still evident in the digit condition (*F*_(1,34)_ = 9.06, *p* < 0.01), with effect size remaining large (*ŋ*^2^ = 0.21), but not in the color condition (*F*_(1,34)_=1.77, *ns*) for which the effect size of group was small (*ŋ*^2^ = 0.05).

### noLD-DD sample

Mean (and SDs) for the three experimental tasks of both noLD-DD participants and respective control group are reported in Table 4 according to type of stimulus.

As for RAN times, a significant group effect was evident only in the digit condition (*F*_(1,26)_ = 5.15, *p* < 0.05): the noLD-DD group performed more slowly than control average readers on RAN of digits. Effect size for the group factor was large (*ŋ*^2^ = 0.16). RAN times for colors were not significantly different between the two groups (*F*_(1,26)_ = 0.96, *ns*), the effect size being small (*ŋ*^2^ = 0.04).

ANOVA on DN response latencies showed significant differences between groups for both digits (*F*_(1,26)_ = 4.06, *p* = 0.05) and colors (*F*_(1,26)_ = 5.52, *p* < 0.05): noLD-DD children were slower than control children with both types of stimulus. Effect sizes were medium-high and large for digits (*ŋ*^2^ = 0.13) and colors (*ŋ*^2^ = 0.17), respectively.

Finally, DR latencies did not differ significantly between noLD-DD and controls, for both digits (*F*_(1,26)_ = 0.06, *ns*) and colors (*F*_(1,26)_ = 0.03, *ns*), with no appreciable effect size of group in both conditions (*ŋ*^2^ = 0.00 for both digits and colors). No difference emerged also for accuracy level (see Section on Data Analyses), ruling out the possibility of a speed-accuracy trade-off in the noLD-DD group.

When differences on DN latencies were partialled out by ANCOVA, differences between the two groups on RAN times for digits disappeared (*F*_(1,26)_ = 1.47, *ns*), with a small effect size of group (*ŋ*^2^ = 0.05).

### LD-DD vs. noLD-DD sample

No significant difference emerged between LD-DD and noLD-DD children, regardless of the experimental task and the type of stimulus (*F*_(1,30)_ = 0.23 and *F*_(1,30)_ = 1.46 for RAN of digits and colors respectively, *F*_(1,30)_ = 2.18 and F_(1,30)_ = 0.39 for DN of digits and colors respectively, *F*_(1,30)_ = 0.03 and *F*_(1,30)_ = 0.12 for DR of digits and colors, respectively).

Effect size of the group was small or absent for all comparisons, with the only exception of DN of digits in which a medium effect size was evident (*ŋ*^2^ = 0.07).

## Discussion

Children with DD were significantly slower than controls on serial RAN task, in both the digit and the color condition. By contrast, accuracy was quite high in both groups. These results are entirely consistent with those from a wide literature documenting deficient RAN speed in subjects with DD in spite of a very low incidence of naming errors (Wolf and Bowers, 1999; Kirby et al., 2010).

DD participants also showed significantly longer latencies than controls on naming items presented in a discrete form. Slowness of DD children on the DN task was evident for both digits and colors and emerged as a robust group effect in both the conditions. This finding confirms a substantial amount of evidence showing slowness of children with DD in naming familiar items even under the simple condition in which items are singularly presented (Bowers and Swanson, 1991; Fawcett and Nicolson, 1994; Chiappe et al., 2002; Jones et al., 2009; Zoccolotti et al., 2013). The typical interpretation offered for this result is in terms of impaired lexical access and/or retrieval from long-term memory (Walsh et al., 1988; Bowers and Swanson, 1991; Pennington et al., 2001). Such interpretation would also be consistent with results from another line of research documenting impaired performance of children with DD on both confrontation naming and naming to definition tasks (for a review see Snowling, 2000).

If a word-retrieval deficit is the reason for delayed vocal reaction times of children with DD on DN tasks, the same deficit could be easily identified as one factor underlying RAN difficulties of these subjects as serial RAN tasks necessarily involve single items naming. Indeed, this is one of the prominent explanations of such difficulties (Torgesen et al., 1997; Pennington et al., 2001; Chiappe et al., 2002). By this reasoning, RAN speed relates to reading performance as the former taps the same efficiency of accessing and retrieving phonological information the latter requires for accurately and effortlessly mapping orthography onto phonology at both lexical and sub-lexical level.

However, naming of single items not only requires lexical access, but also visual recognition of the item to be named. Thus, interpretation of impaired performance of children with DD on both DN and (at least partially) RAN tasks in terms of a name-retrieval deficit remains speculative, although plausible, until a deficit of visual processing on DN performance can not be excluded.

To this aim, in the present study we introduced a motor choice-reaction time task using the same singularly presented stimuli as in the DN task, where participants had to discriminate between a target stimulus and four distracters. Results on this task did not discriminate between children with DD and typical reader controls; response latencies in our DR task were almost the same in the two groups, regardless of type of stimulus. No statistical difference emerged also for level of response accuracy, so leaving out a speed-accuracy trade-off possibility in the performance of participants with DD. Then, our results do not support the hypothesis that some early visual deficit in single item recognition subtends deficient performance of DD subjects on DN tasks and consequently their reduced speed also on RAN tasks. To the best of our knowledge this is the first time a control on visual-perceptual factors underlying performance on DN task is made using the same stimulus materials as in such task.

Results discussed up to now support the role of a phonological-retrieval deficit in explaining slowed response latencies in the DN tasks, and possibly also reduced speed in RAN tasks, in subjects with DD. However results in our DD sample indicate that other mechanisms underpinning RAN performance contribute in mediating its relationship with reading. In fact, when influence of DN tasks latencies on RAN speed was controlled by covariance analysis, differences between DD and normal readers on RAN survived remaining robust for the digit condition, while disappearing for the colors condition. The unique contribution of serial RAN tasks to reading performance over that played by discrete-trial format of naming tasks is well documented in the literature (Bowers and Swanson, 1991; Jones et al., 2009; Logan et al., 2011; Georgiou et al., 2013; Zoccolotti et al., 2013).

Several aspects of serial RAN tasks might account for the greater differentiation of reader groups by this measure compared to that by discrete trial measures. One of these aspects refers to oculomotor requirements for efficient left-to-right visual scanning of stimuli presented in a matrix format, very similar to those necessary for efficient reading of texts. Another putative mechanism has been identified in attentional processes pertinent to the managing of serial information, especially those underlying parafoveal processing of upcoming items, consequent saccadic preparation, eye-movement execution and subsequent articulating of speech output. In a recent study where total response times (from the stimulus onset to the end of its pronunciation) were recorded, Zoccolotti et al. (2013) found that typical readers were significantly faster reading words arranged in rows than singly displayed words, at odds with participants with DD who had a disadvantage in reading multiple stimuli for long words. This last result is in line with other evidence documenting deficient parafoveal processing in subjects with DD (Chace et al., 2005; Jones et al., 2009, 2013). Interpretations of RAN deficits in terms of managing serial information are also reminiscent of another longstanding theory of RAN underpinnings proposed by Wolf, Bowers and colleagues (Bowers, 1995; Wolf and Bowers, 1999; Wolf et al., 2000). According to Bowers (1995) “*…although rapide/precise timing mechanisms may underpin performance on all the naming speed measures, the serial format for naming speed requires additional coordination of processes used to extract information from serial visual arrays*” (p. 211–212), thus making RAN tasks more similar to reading than DN naming tasks.

In our study, the unique contribution of serial RAN over that of DN in discriminating between DD and average readers was evident for the digits, but not for the colors, condition. Indeed, this pattern of results seems consistent with the interpretations which emphasize the role of processes pertaining to management and integration of multiple sub-processes in mediating RAN-reading relationship. A stronger predictive role of RAN of alphanumeric stimuli than non-alphanumeric stimuli over reading is not uncommon in the RAN literature (Walsh et al., 1988; McBride-Chang, 1996; Schatschneider et al., 2004). The usual explanation is that digits (like letters) constitute highly constrained categories that can be processed more “automatically” with practice than colors and figures (Wolf and Bowers, 1999). In turn, faster naming for alphanumeric than non-alphanumeric stimuli would let the integration of multiple sub-components involved in serial RAN to occur more efficiently with the former (Protopapas et al., 2013), also making RAN of letters and digits a closer approximation to fluent reading.

In the present study we treated RAN times as a unitary measure, not distinguishing between times to articulate each item and duration of pauses between subsequent articulations. As a consequence, another possible explanation for the unique contribution of serial RAN tasks to reading performance over that played by discrete-trial format might be that a lower articulation rate of participants with DD with respect to controls would selectively lengthen RAN times in the former without affecting response latencies in the DN tasks. However, various considerations make this hypothesis unlikely. First, although exceptions exist (e.g., Georgiou et al., 2008), recent investigations of the RAN components have mostly agreed that inter-item pauses and not articulation times are significantly related to reading (Neuhaus et al., 2001; Georgiou et al., 2006; Araújo et al., 2011b). Second, in a recent study where vocal reaction times and pronunciation times during a single-item reading task were measured, longer pronunciation times of DD with respect to control readers were found only for non-words and words exceeding six letters (Martelli et al., 2014). However, in our study words to be articulated for the naming tasks were all very high frequency words and shorter than six letters. Moreover, in a previous study on Italian second graders (unselected for reading ability) the association between articulation rate and reading speed of a text (which includes pronunciation times) was virtually absent; likewise, the contribution of RAN total times to reading speed of text remained significant and substantially unchanged after controlling for articulation rate (Gasperini et al., 2008).

The pattern of results described above refers to the whole sample of children with DD. Did this pattern hold when analyses were run separately on the two subgroups with (LD) or without (noLD) a history of previous LD? In the last years a number of studies has provided evidence for a partially different behavioral and neurocognitive profile between LD- and noLD-DD children (Brizzolara et al., 2006; Scuccimarra et al., 2008; Chilosi et al., 2009; Pecini et al., 2011). Consistently with such evidence, we expected a concurrent language weakness in the former group, which might have a particularly relevant impact on the DN performance of DD children with LD. Indeed, this is what we found. The LD-DD children scored significantly lower than the noLD-DD children in measures of phonological processing (PSTM), verbal semantic knowledge and written text comprehension, confirming our previous results. Moreover, although both DD groups were slower compared to typical readers on the DN task, naming single items proved to be more difficult for LD- than noLD-DD children, as indicated by the more marked effect size for in LD than in noLD participants DD ( in the comparison with the control group) for both digits and colors. Indeed, a possible stronger lexical access deficit in the LD- than in noLD-DD group had been anticipated. However, such a deficit was not accounted for by a semantic retrieval impairment in the former group, in addition to a phonological retrieval deficit shared by both DD groups, as we had hypothesized. In fact, the DN deficit of the LD-DD children was not more evident for colors than for digits, as it would be expected if a semantic impairment would underlie the lexical access deficit of the LD-DD children. One possible explanation for the more marked lexical access difficulties of these subjects might well be only in terms of a phonological retrieval deficit, which would be more pronounced for the LD-DD children. Such an explanation would be consistent with data showing poorer phonological processing abilities in children with SLI than in children with DD without oral language problems (Kamhi and Catts, 1986; Tallal et al., 1997).

By contrast, both LD- and noLD-DD groups were indistinguishable from controls on the DR task, regardless of the type of stimulus, on both response latencies and accuracy.

Overall, these results indicate that children with DD have a discrete-item naming deficit which cannot be accounted for by a visual-perceptual impairment, but needs to be explained as a name-retrieval deficit. Such a deficit, is more marked in DD children with a previous LD and a concomitant verbal weakness.

Also other differences between LD- and noLD-DD children emerged in our study. A different pattern of results was in fact evident in the two groups when analyzing data of the RAN task. First, while for LD-DD children differences from controls were present regardless of the type of stimulus, for noLD-DD children impaired RAN speed occurred only for digits. Moreover, while a significant slowness of LD-DD children with respect to controls on RAN task survived for digits (but non for colors) after controlling for differences in DN response latencies, no significant difference was still evident on RAN of digits between noLD-DD and control readers when DN speed was controlled.

The absence of a “specific” serial RAN deficit for colors in both DD groups is consistent with the hypothesis of a general reduced role of processes pertaining to the managing of multiple activities in RAN of non-alphanumeric stimuli (Protopapas et al., 2013). However, a “non-specific” RAN deficit for colors was still evident in LD-DD children, possibly as a consequence of their marked name-retrieval deficit for this type of stimuli, which would “propagate” to the serial condition. The same would not occur in the noLD-DD group, who showed a much smaller deficit of lexical access for colors with respect to LD children, with the result that noLD-DD children did not differ significantly from controls on RAN of colors.

The difference between the two DD groups was however more relevant on RAN of digits, in which both samples were impaired, but only in the LD children significant slowness with respect to controls survived after controlling for differences in response latencies in the DN task. This finding seems indicative of a greater difficulty of LD- with respect to noLD-DD children in rapidly and precisely integrating different cognitive and linguistic processes underlying RAN performance with digits. On the basis of our data reasons for a different involvement of “synchronization” deficits in the two DD subtypes cannot be much more than speculative. It might be that the larger name-retrieval deficit of LD- than noLD-DD children is responsible for a greater impairment of precise/timing mechanisms in the former than in the latter. When lexical access severely taxes the processing capacity of the child, in fact, integrating such a process with other activities is also severely affected. However, also a specific deficit with a precise/timing mechanism and/or sequential processing *per se* might affect LD DD children, accounting for the unique contribution of serial RAN over the one by discrete naming in differentiating this group from controls.

It remains unclear why noLD-DD subjects showed a “non-specific” RAN deficit for digits, but not for colors; their lexical access deficit was in fact very similar for the two types of stimulus. An explanation for such difference in terms of a reduced efficiency of noLD-DD children in simultaneously performing multiple activities, which would mainly occur for digits rather than colors, seems unlikely; not only a statistically significant difference of these subjects from controls on RAN of digits was not more evident once differences on DN of the same material was controlled for, but the group effect size *per se* (as resulted from the covariance analysis) was small.

Results of our study were obtained on groups of subjects widely ranging in school grade (from second to eighth). Such heterogeneity might raise some doubts on the interpretation of results concerning cognitive underpinnings of RAN speed deficit in children with DD. However, when we compared absolute speed of reading (syllables/s in reading of lists of single words) between the subgroup of Primary school children (from second to fifth grade) and the subgroup of Secondary school children (from sixth to eighth grade) no significant difference emerged. The absolute reading speed level of both subgroups was comparable with that of Italian second grade typical readers, as provided by the norms of the standardized reading test employed (Sartori et al., 2007). At such level, Italian normal readers are in a stage in which they are shifting from sub-lexical (serial) to lexical (holistic) written word processing (Zoccolotti et al., 2005; Orsolini et al., 2006) and, consequently, the need for a rapid access to lexical phonology becomes crucial. Indeed, according to some authors, the characteristic reading speed deficit of Italian children with DD would mainly reflect a problem in acquiring efficient use of a lexical strategy in reading (Zoccolotti et al., 1999; Orsolini et al., 2009). However, recently Zoccolotti et al. (2013) highlighted a further *locus* for the impaired reading fluency of Italian DD in a reduced advantage with multiple over single words in reading of these subjects with respect to average readers. Both of these impairments (lexical-reading and simultaneous processing of multiple items) are consistent with deficits in lexical access and in managing the sequential information in serial RAN for DD children, as suggested by our results.

One limitation of the present study is that evidence of LD in the pre-school years is retrospective, but this was unavoidable as the children with DD were referred to us for assessment of academic achievements at school age. Such limitation might raise some doubts as to the reliability of classification of participants with DD according to whether or not they had a history of previous LD. However, we also found *concomitant* weaker verbal abilities of the LD- than the noLD-DD group, not limited to deficient oral phonological processing but also encompassing impaired verbal semantic knowledge and text reading comprehension, despite absence of differences in reading decoding abilities.

Another weakness of our study is the relatively small size of each group with DD, when DD participants were subdivided according to the presence or absence of a previous LD. In these conditions significant, but not strong enough, effects of both reading group and type of stimulus on performance in the different experimental tasks might not emerge because of low statistical power. Then, future studies should address issue of cognitive deficits underpinning RAN impaired performance of different categories of DD subjects using larger sample sizes for each subgroup. It should be noted, however, that in our study analyses of the group effect size on the experimental measures turned out to be small at best for those comparisons which were not statistically significant. Then, a substantial change of our results with larger samples does not seem very likely.

Despite the above limitations, we think our study offers a contribution to a better understanding of the reasons why RAN speed discriminates between DD and typical readers, as well as in pointing out possible different cognitive mechanisms at the basis of RAN impairment in different subgroups of children with DD. Recently, there has been a growing interest for potential differences in cognitive processes underpinning RAN performance in different populations of subjects. However, up to now this interest has mainly concentrated on identifying possible developmental differences in the pattern of interrelations among different naming paradigms and/or measures and reading in samples of mostly typically developing readers in different school grades (e.g., Georgiou et al., 2006; de Jong and Messbauer, 2011; Protopapas et al., 2013). In the present study we expanded such aim by comparing different subtypes of children with DD, classified according to whether or not they had a history of previous language delay, which represent two partially different neurocognitive phenotypes. Results from this comparison indicate that this is a worth pursuing goal and a potentially fruitful area of research, as superficially similar RAN impairments in different populations of subjects with DD may obscure at least partially different underlying cognitive deficits.

## Authors’ contribution

Filippo Gasperini, Child Neuropsychologist, PhD, contributed to the design of the work, acquisition, analysis, and interpretation of the data, to writing the paper and final approval of the version to be published.

Daniela Brizzolara, Child Neuropsychologist, contributed to the design of the work, to interpretation of the data and to writing the paper, revising the paper critically for intellectual content and final approval of the version to be published.

Paola Cristofani, Child Psychologist, contributed to acquisition and analysis of experimental data and final approval of the version to be published.

Claudia Casalini, Child Psychologist, contributed to acquisition of clinical data, revising the final manuscript and final approval of the version to be published.

Anna Maria Chilosi MD, PhD, Specialist in Child Neurology and Psychiatry, contributed to acquisition of anamnestic data of all children with DD, to writing, revising the paper critically for intellectual content and final approval of the version to be published.

## Conflict of interest statement

The authors declare that the research was conducted in the absence of any commercial or financial relationships that could be construed as a potential conflict of interest.
